# The mediating role of resilience in the relationship between cognitive flexibility and psychological well-being in patients with irritable bowel syndrome: a cross-sectional study

**DOI:** 10.1186/s12876-026-04654-3

**Published:** 2026-01-31

**Authors:** Mohammad Reza Tamannaeifar, Zahra Shirani, Mahboobe Esmikhani, Zeinab Zaremohzzabieh

**Affiliations:** 1 Department of Psychology, Faculty of Humanities, University of Kashan, Kashan, Iran; 2 Women and Family Studies Research Center, University of Religions and Denominations, Qom, Iran; 3 Department of Psychology, Faculty of Economic and Social Sciences, Buali Sina University, Hamadan, Iran

**Keywords:** Cognitive flexibility, Irritable bowel syndrome, Psychological well-being, Resilience

## Abstract

**Background:**

Irritable bowel syndrome (IBS) is one of the most common brain–gut interaction disorders, characterized by chronic abdominal pain, altered bowel habits, and heightened stress responses that significantly affect patients’ psychological functioning and quality of life. Given that chronic stress plays a central role in the onset and maintenance of IBS symptoms, identifying psychological resources that promote adaptation and well-being is crucial. The present study aimed to examine the mediating role of resilience in the relationship between cognitive flexibility and psychological well-being among patients with IBS.

**Method:**

This descriptive-correlational study employed a structural equation modeling approach. The statistical population included all IBS patients who referred to the Gastrointestinal Health Center at Al-Zahra Hospital in Isfahan in 2024. A sample of 300 patients was selected using convenience sampling. Data were collected using the Psychological Well-Being Scale, Cognitive Flexibility Inventory, and Connor–Davidson Resilience Scale, and analyzed using SPSS and AMOS version 28.

**Findings:**

Results showed that cognitive flexibility and resilience significantly predicted psychological well-being, and that resilience mediated the relationship between cognitive flexibility and psychological well-being. These findings highlight the importance of resilience and cognitive flexibility as protective psychological resources that help patients manage stress and improve well-being in the context of IBS.

## Introduction

Irritable bowel syndrome (IBS) is a common disorder characterized by dysfunction in the brain–gut interaction [1]. It manifests as recurrent abdominal pain and abnormal bowel patterns, including diarrhea, constipation, or both. Several pathophysiological mechanisms have been proposed, such as changes in gut motility, impaired barrier function, immune system activation, visceral sensitivity, and abnormalities within the central nervous system [2]. These mechanisms are influenced by genetic predisposition, stress, antibiotic use, infections, diet, and psychological distress [3]. IBS is highly prevalent worldwide, affecting approximately 10–20% of the population [4], while its prevalence in Iran is estimated at 5.8% [5]. Importantly, IBS disrupts daily functioning and reduces quality of life by interfering with daily activities, increasing health anxiety, creating an economic burden, and promoting food avoidance [6].

Evidence shows that gastrointestinal symptoms in IBS worsen with increased psychological distress and improve when psychological indicators improve [7, 8]. Moreover, worsening symptoms heighten visceral sensitivity and diminish quality of life [9]. Psychological constructs such as catastrophizing, symptom hypervigilance, avoidance, stress, pessimism, neuroticism, and emotional suppression have been shown to exacerbate and maintain IBS symptoms [10]. Consequently, people with IBS often report lower psychological well-being, including reduced resilience, positive affect, self-efficacy, and emotion regulation, compared with healthy individuals, which is linked to poorer physical and mental health as well as diminished quality of life [11]. Supporting this, Alshaikh et al. [12] found that higher pain, anxiety, and depressive symptoms are associated with lower psychological well-being, prompting experts to recommend integrating psychological interventions alongside medical care.

Among these psychological factors, cognitive flexibility and resilience have received growing attention. Psychological well-being has been consistently linked to better health outcomes, such as reduced pain and fewer physical symptoms, in both healthy and chronically ill populations [13, 14]. According to Ryff and Keyes’ [15] model, psychological well-being involves traits such as self-acceptance, purpose in life, personal growth, environmental mastery, and positive relationships with others. Cognitive flexibility and resilience are particularly important for maintaining these outcomes in chronic illnesses like IBS.

Cognitive flexibility refers to the ability to shift mental frameworks and strategies in response to changing circumstances [16]. High cognitive flexibility is associated with better adaptation, problem-solving, and adjustment to stressors [17, 18], while low flexibility has been linked to poorer quality of life and more severe IBS symptoms [19–22]. Resilience, in turn, refers to the ability to adapt effectively to adversity and recover from stress [23, 24]. Studies show that IBS patients generally have lower resilience compared with the general population, which contributes to more severe symptoms and poorer mental health [25]. Importantly, prior research suggests that cognitive flexibility supports resilience, and together they play a role in promoting psychological well-being [26–30].

However, despite this evidence, limited research has examined how resilience functions as a mediating mechanism between cognitive flexibility and psychological well-being in IBS patients. While previous studies have demonstrated independent associations among these variables, the specific indirect pathway remains underexplored [20, 29, 31–35]. Clarifying this relationship can contribute to the design of psychological interventions that strengthen resilience and enhance psychological well-being in IBS patients.

Accordingly, the present study aims to investigate the mediating role of resilience in the relationship between cognitive flexibility and psychological well-being in patients with IBS. This focus on resilience as a mediator offers a novel perspective by extending prior work beyond simple associations, thereby providing deeper insight into psychological mechanisms relevant for intervention development.

## Methodology

### Study design

A cross-sectional survey design was employed to examine the relationships among cognitive flexibility, psychological well-being, and resilience in patients with IBS. This design is commonly used to investigate associations between psychological constructs at a single point in time [36].

### Participants and data collection

The statistical population included all patients diagnosed with IBS at the Gastrointestinal Health Center of Alzahra Hospital in Isfahan in 2024. Although there is no consensus on the required sample size for factor analysis and structural equation modeling, Kline [37] suggested a minimum sample of 200 participants. Therefore, based on Kline’s recommendation and accounting for potential dropouts, a sample of 300 individuals was selected to ensure sufficient statistical power.

Participants were recruited through random sampling [38]. Data were collected in a single session using self-administered questionnaires. Potentially eligible participants were approached at the center, informed about the study’s objectives and procedures, and provided written informed consent. The questionnaires were completed in a private room to ensure confidentiality and required approximately 20–30 min to complete. Screening for eligibility was conducted through brief self-report questions and review of available medical records at the time of recruitment.

### Inclusion and exclusion criteria

The inclusion criteria were: (a) age between 18 and 65 years; (b) a diagnosis of IBS confirmed by a gastroenterologist according to the Rome IV diagnostic criteria, which required recurrent abdominal pain occurring at least one day per week on average over the past three months, associated with two or more of the following: related to defecation, associated with a change in stool frequency, or associated with a change in stool form [39, 40]; (c) a minimum symptom duration of six months; and (d) fluency in Persian.

Exclusion criteria included: (a) the presence of organic gastrointestinal disease; (b) a history of major abdominal surgery; (c) self-reported diagnosis of severe, untreated psychiatric disorders, such as psychotic disorders, bipolar disorder, or active substance use disorder, defined as conditions associated with marked cognitive impairment, active psychotic symptoms, or loss of reality testing that could compromise the ability to provide informed consent or to validly complete self-report measures; and (d) the presence of alarm symptoms (e.g., unexplained weight loss, rectal bleeding).

Importantly, individuals with depression or anxiety were not excluded unless symptoms were severe and untreated to the extent that they impaired informed consent or questionnaire completion. Based on these criteria, a small number of individuals (*n* = 6) were excluded at the screening stage due to severe psychiatric conditions. It is acknowledged that this exclusion criterion may limit the generalizability of the findings to the broader IBS population, which has a high prevalence of comorbid psychological conditions such as anxiety and depression [41].

### Research instruments

Three validated instruments were used to measure the study variables.

Psychological Well-Being Scale (PWBS) developed by Ryff [42], this scale consists of 18 items measuring six components: environmental mastery, self-acceptance, positive relations with others, purpose in life, personal growth, and autonomy. Items are rated on a 6-point Likert scale ranging from “strongly disagree” (1) to “strongly agree” (6). Higher scores indicate greater psychological well-being. The scale’s concurrent validity was supported by significant correlations (*r* = 0.29–0.62, *p* < 0.01) with Rosenberg’s [43] Self-Esteem Scale. Reliability was originally reported as α = 0.92 [44]. In the Iranian adaptation, confirmatory factor analysis indicated good model fit (CMIN/DF = 1.68–1.94, GFI = 0.92–0.96, RMSEA = 0.014–0.045), with Cronbach’s alphas across subscales ranging from 0.46 to 0.78 [45]. In the present study, Cronbach’s alpha was 0.95, indicating excellent internal consistency.

Cognitive Flexibility Inventory (COFI) developed by Dennis and Vander Wal [46], this 20-item inventory measures two components: problem-solving processing and perception of controllability. Items are rated on a **7**-point Likert scale (1 = strongly disagree to 7 = strongly agree), with six items reverse-scored (2, 4, 7, 9, 11, and 17). The original reliability coefficients were 0.84, 0.91, and 0.91 for the subscales and total scale, respectively. Concurrent validity with the Beck Depression Inventory-II [47] yielded *r* = − 0.39 (*p* < 0.01) [48]. In Iran, exploratory and confirmatory factor analyses confirmed the two-factor structure (χ²/df = 2.059, AGFI = 0.854, CFI = 0.885, RMSEA = 0.066), with Cronbach’s alphas of 0.89 (problem-solving processing), 0.78 (controllability), and 0.81 (total) [49].

In this study, Cronbach’s alpha was 0.89.

Connor-Davidson Resilience Scale (CD-RISC-10) developed by Campbell-Sills and Stein [50], the 10-item short form assesses the ability to cope with stress. Items are rated on a 5**-**point Likert scale (0 = strongly disagree to 4 = strongly agree), with total scores ranging from 0 to 40; higher scores indicate greater resilience. The developers reported α = 0.85 and good construct validity (χ²/df = 1.404, CFI = 0.999, RMSEA = 0.027) [39]. In this study, Cronbach’s alpha was 0.82, confirming acceptable reliability.

### Validation and data processing

Before analysis, the collected data were screened for missing and inconsistent responses. Incomplete responses were defined as cases with more than 10% missing items in any questionnaire and were excluded from further analysis. Cases with less than 10% missing data were retained, and the missing values were addressed using the regression imputation method, which estimates missing values based on predictive relationships among observed variables. This approach is recommended for minimizing bias and preserving data variability when the proportion of missing data is low [51]. The missing data rate across all items was below 2%, indicating high data quality. All psychometric instruments used had been previously validated in Iranian samples. Cronbach’s alpha coefficients were recalculated in this study to confirm internal consistency reliability.

### Ethical considerations

The study was conducted after receiving approval from the Ethics Committee of Kashan University (No. KA-2025-102) and the administration of Alzahra Hospital, Isfahan. All procedures adhered to the ethical standards of the institutional and national research committees and the 1964 Helsinki Declaration [52] and its later amendments, or comparable ethical standards. Participants were fully informed about the study’s objectives and their right to withdraw at any time, and written informed consent was obtained from all participants. As this was an observational, non-interventional study, it was not registered in a public clinical trial registry.

### Data analysis

Preliminary analysis and missing data were determined using the Statistical Package for the Social Sciences (SPSS, version 26; IBM Corp., Armonk, NY, USA). Descriptive statistics (means, standard deviations, and frequency distributions) were calculated to summarize the demographic and main study variables. To investigate relationships between variables, Pearson correlation coefficients were computed. Furthermore, to test the proposed model and examine the structural relationships between variables, structural equation modeling (SEM) was employed using AMOS software (IBM Corp., Armonk, NY, USA). The model fit was evaluated by common fit indices, including χ²/df, GFI, CFI, and RMSEA. Cronbach’s alpha coefficients were also calculated to assess the internal consistency and reliability of all scales used in the study.

## Results

### Descriptive statistics

First, the demographic characteristics of the participants were examined. In this study, out of the total sample, 231 individuals (77%) were women and 69 individuals (23%) were men. Regarding education levels, 9 participants (3%) had a high school diploma, 97 (32.1%) held a diploma, 15 (5%) had an associate degree, 103 (34.1%) had a bachelor’s degree, 63 (20.9%) had a master’s degree, and 12 (4%) held a doctoral degree. In terms of marital status, 196 participants (65.33%) were married, and 53 (35.33%) were single. The average age of the participants was 29.31 years, with a standard deviation of 9.859.

Next, the descriptive statistics and normality of the research variables were examined. Table 1 presented the number of participants, means, standard deviations, skewness, and kurtosis for each variable in the study.

**Table 1:** Descriptive statistics and normality of the study

No.	Constructs	*N*	Mean	SD	Skewness	Kurtosis
1	Environmental Mastery	300	9.87	4.84	-0.12	-1.40
2	Self-Acceptance	300	9.38	4.37	-0.18	-1.63
3	Positive Relations with Others	300	9.75	3.99	0.05	-1.30
4	Purpose in Life	300	9.24	5.07	0.51	-0.91
5	Personal Growth	300	10.53	5.02	0.14	-1.20
6	Autonomy	300	10.28	4.67	0.13	-1.21
7	Total Psychological Well-Being Score	300	59.05	25.95	-0.11	-1.25
8	Problem-Solving Processing	300	66.40	13.13	0.95	0.38
9	Perceived Controllability	300	24.16	8.49	0.54	-0.25
10	Resilience	300	29.46	9.84	0.18	1.15

Note. Multivariate normality: Mardia’s coefficient = 0.91, Critical ratio = 0.82

To perform SEM using parametric methods, several assumptions needed to be met. One important assumption was the normality of the data and variables. Both numerical indices and visual inspections were used to evaluate univariate normality. Specifically, skewness and kurtosis values between − 2 and + 2 were considered acceptable indicators of normal distribution [53]. As shown in Table 1, the skewness and kurtosis values for all variables fell within this acceptable range. Histograms also appeared approximately symmetric and bell-shaped, confirming univariate normality.

Multivariate normality was assessed using the standardized Mardia’s [54] coefficient and critical ratio. Values below 5 indicate normal multivariate distribution [55]. In this study, Mardia’s coefficient (0.91) and critical ratio (0.82) confirmed multivariate normality. Independence of errors was assessed using the Durbin–Watson test, which yielded values between 1.5 and 2.5, indicating independence. Finally, to ensure the absence of multicollinearity, tolerance and variance inflation factor (VIF) statistics were examined. None of the tolerance values were below 0.1, and all VIF values were under 10, confirming no multicollinearity.

### Correlations

As shown in Table 2, the study variables demonstrated moderate to high positive intercorrelations, particularly between resilience, problem-solving processing, perceived controllability, and psychological well-being. While the correlations were statistically significant, they did not indicate redundancy among constructs. According to Cohen [56], correlations between 0.50 and 0.70 reflect substantial but not problematic relationships, suggesting theoretical relatedness rather than conceptual overlap. Theoretically, these constructs are distinct: problem-solving processing and perceived controllability reflect cognitive appraisals and coping processes, resilience reflects adaptive capacity, and psychological well-being represents an outcome of sustained adaptation.

**Table 2:** Correlation matrix of the research variables

No.	Constructs	1	2	3	4
1	TPW	1			
2	PSP	0.58^**^	1		
3	PC	0.47^**^	0.29^**^	1	
4	RS	0.78^**^	0.64^**^	0.63^**^	1

Note. *TPW* Total Psychological Well-being Score, *PSP* Problem-Solving Processing, *PC* Perceived Controllability, *RS* Resilience

All correlations are significant at the 0.01 level

Discriminant validity was assessed to ensure empirical distinctiveness among constructs. The square roots of the average variance extracted (AVE) for each construct exceeded their inter-construct correlations, confirming discriminant validity [57]. VIF values ranged from 1.42 to 2.38, and tolerance values exceeded 0.40, indicating the absence of multicollinearity (Hair et al., 2019).

### Model fit indices

The fit indices of the research model were presented in Table 3.

**Table 3:** Fit indices of the proposed research model

Type of Index	Index	Value	Acceptable Value
Absolute Indices	CMIN	62.20	—
	df	24	—
	CMIN/df	2.59	< 3
	p-value	0.001	—
Relative Indices	RMSEA	0.045	< 0.08
	PCLOSE	0.001	—
	CFI	0.935	> 0.90
	AGFI	0.936	> 0.90
	PCFI	0.611	> 0.60
	PNFI	0.618	> 0.60
	IFI	0.930	> 0.90
	GFI	0.912	> 0.90
	NFI	0.996	> 0.90

Note. *CMIN* Normalized Chi-Square, *df* Degrees of Freedom, *RMSEA* Root Mean Square Error of Approximation, *PCLOSE* P-value for closeness of fit, *CFI* Comparative Fit Index, *AGFI* Adjusted Goodness of Fit Index, *PCFI* Parsimonious Comparative Fit Index, *PNFI* Parsimonious Normed Fit Index, *IFI* Incremental Fit Index, *GFI* Goodness of Fit Index, *NFI* Normed Fit Index

To test the proposed model in the current study, SEM was applied. To evaluate the model’s fit, the fit indices presented in Table 5 were used. Specifically, if the NFI, NNFI, CFI, IFI, GFI, and AGFI were all greater than 0.90, and the PCFI and PNFI were above 0.60, these indicated a good and acceptable model fit. According to the results of the final research model, as can be seen, all of these indices were within the desirable range. Additionally, if the value of the RMSEA was less than 0.08, it also indicated a good model fit [53]. In this study, the significance level for the PCLOSE index was 0.001, and the RMSEA value was 0.045, which according to Klein’s [37] model confirmed that the research model fits well.

### Common method bias and latent common factor tests

To address potential concerns regarding a common latent explanatory factor, both Harman’s single-factor test and a latent common method factor (CMF) analysis were conducted. First, Harman’s single-factor test revealed that the first unrotated factor accounted for 32.4% of the total variance, which is well below the 50% threshold, suggesting that common method bias was not a major concern in this dataset [58].

Second, a latent common method factor analysis was performed by including a single unmeasured method factor in the confirmatory factor analysis (CFA), allowing all observed indicators to load onto both their theoretical constructs and the latent method factor. The inclusion of this latent factor produced only a marginal improvement in model fit (ΔCFI = 0.008; ΔRMSEA = 0.003), and all substantive path coefficients remained significant and of similar magnitude to those in the original model. Table 4 summarizes the results of the CFA models with and without the latent method factor.

**Table 4:** Model fit comparison: with and without latent common method factor

Model	CFI	RMSEA	ΔCFI	ΔRMSEA
Without CMF	0.935	0.045	—	—
With CMF	0.943	0.042	0.008	0.003

These findings indicate that the correlations among constructs are not driven by a single latent common factor, supporting the distinctiveness of the theoretical constructs—cognitive flexibility, resilience, and psychological well-being. The results strengthen confidence that the relationships identified in the model reflect true psychological associations rather than methodological artifacts.

### Structural equation modeling results

As shown in Table 5, the standardized and direct coefficients for problem-solving processing (*β* = 0.62, *p* = 0.001), perceived controllability (*β* = 0.49, *p* = 0.001), and resilience (*β* = 0.48, *p* = 0.002) had a direct and significant effect on psychological well-being.

**Table 5:** Standardized and direct coefficients in the fitted research model

Paths	β	S.E	t	sig
PSP → TPW	0.56	0.21	2.26	0.023
PC → TPW	0.62	0.33	3.34	0.017
RS → TPW	0.79	0.39	19.16	0.001

Note. *TPW *Total Psychological Well-being Score, *PSP* Problem-Solving Processing, *PC* Perceived Controllability, *RS* Resilience

### Mediation analysis

To determine the significance of cognitive flexibility on psychological well-being through the mediating role of resilience, the bootstrap method was used. According to the results presented in Table 6, if both the lower and upper bounds of the test are either positive or negative and zero does not fall between these two limits, then the indirect causal path was considered significant. Based on the results in Table 4, the indirect effect of cognitive flexibility on psychological well-being through resilience was significant. When both the lower and upper bounds of the 95% confidence interval exclude zero, mediation is supported. Both mediation paths met this criterion.

**Table 6:** Bootstrap results for cognitive flexibility with resilience mediation on psychological Well-Being

Paths	Standardized Indirect Coefficients		Sig.
	LL	UL	
PSP → RS → TPW	0.012	0.164	0.012
PC → RS → TPW	0.023	0.153	0.024

Note. *TPW* Total Psychological Well-being Score, *PSP* Problem-Solving Processing, *PC* Perceived Controllability, *RS* Resilience, *LL* Lower limit, *UL* Upper limit

**Fig. 1:**
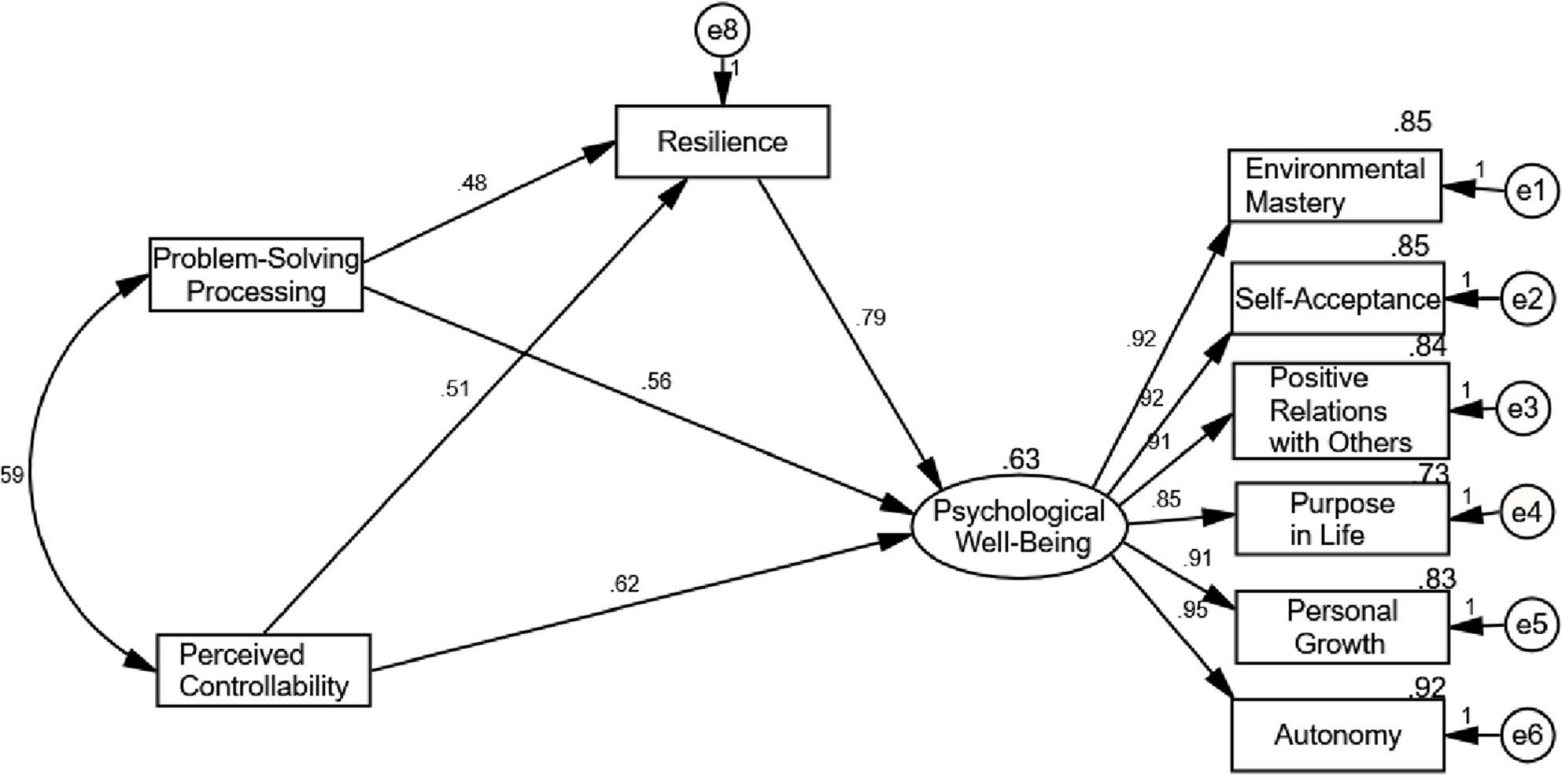
Structural model

Figure 1 presents the structural and final model of the research. The explained variance for the criterion variable (psychological well-being), based on cognitive flexibility with the mediating role of resilience, was found to be 63%. This indicated that cognitive flexibility, through the mediation of resilience, collectively explained 63% of the variance in the psychological well-being of patients diagnosed with IBS.

## Discussion

The aim of this study was to investigate the mediating role of resilience in the relationship between cognitive flexibility and psychological well-being in patients with IBS using structural equation modeling. The findings suggest that resilience serves as a mediator between cognitive flexibility and psychological well-being. Although no previous study has specifically examined resilience as a mediator in this relationship among IBS patients, our results are consistent with prior research that has reported a significant positive relationship between cognitive flexibility and psychological well-being [41, 59–61]. These findings also align with the results of Parker et al. [25] and Hyman et al. [62], who demonstrated a link between resilience and psychological well-being.

Individuals with higher cognitive flexibility are better able to regulate their thoughts and behaviors in response to changing environmental demands. They engage more constructively with challenges and adapt more readily to change. Cognitive flexibility encompasses key abilities such as problem-solving, information processing, attentional control, working memory, positive thinking, and critical thinking, all of which contribute to effective adaptation under stress [63]. These cognitive resources allow individuals to adjust their responses when facing uncertainty or emotionally demanding situations. Importantly, these abilities do not operate in isolation; rather, they shape how individuals appraise stressors and their perceived capacity to manage them. Through flexible problem-solving and cognitive reappraisal, individuals are more likely to perceive stressful situations as manageable and controllable, which forms a core psychological foundation of resilience.

In the context of chronic illness, resilience reflects the capacity to recover from and adapt positively to illness-related stressors. Patients with greater cognitive flexibility tend to tolerate uncertainty more effectively and employ adaptive strategies to process new information, make informed decisions, and reduce anxiety associated with their condition [50]. Flexible problem-solving enables patients to generate alternative coping strategies when symptoms fluctuate, while enhanced perceived controllability over illness-related challenges fosters confidence in one’s ability to cope. These processes collectively strengthen resilience, rather than exerting independent effects on well-being. Moreover, cognitive flexibility supports emotional regulation by enabling individuals to shift attention away from maladaptive thoughts toward more adaptive coping responses, thereby enhancing emotional balance and resilience. This adaptability can lead to better quality of life during and after treatment [3].

Cognitive flexibility also allows patients to reassess life priorities, revise personal goals, and maintain an optimistic outlook, which further strengthens resilience when confronting the ongoing challenges of chronic illness [64]. From this perspective, problem solving and perceived controllability function as mechanisms through which cognitive flexibility is translated into resilient adaptation, rather than as parallel pathways directly influencing psychological well-being. Collectively, these processes highlight cognitive flexibility as a critical psychological resource that contributes to resilience and sustained psychological adaptation.

Resilience, in turn, can positively influence physical health, as chronic stress and anxiety may lead to physical problems, while the ability to cope effectively with stress can improve psychological well-being. Therefore, patients with higher resilience tend to experience less distress and greater psychological well-being [65, 66]. Resilient individuals are better able to mobilize problem-solving skills, maintain a sense of control, and recover emotionally following symptom exacerbations, which helps buffer the negative psychological impact of chronic illness. They are better able to solve problems, adapt to change, and maintain positive emotions [25, 67]. Consequently, resilience promotes life satisfaction, emotional stability, and happiness [68]. Enhancing resilience therefore supports effective stress management, which contributes directly to psychological well-being [24, 26].

## Conclusion

IBS is a prevalent disorder of brain–gut interaction that substantially affects patients’ psychological functioning and quality of life. The findings of the present study suggest that cognitive flexibility contributes to psychological well-being in patients with IBS both directly and indirectly through the mediating role of resilience. Patients with higher cognitive flexibility are better equipped to manage anxiety, health-related symptoms, and stress, leading to improved emotional adjustment and reduced psychological distress.

Conversely, low cognitive flexibility diminishes resilience, heightens stress sensitivity, and reduces coping capacity. Thus, cognitive flexibility influences the psychological well-being of IBS patients through the mediating effect of resilience. From a clinical perspective, the interaction among emotional, cognitive, and behavioral factors particularly cognitive flexibility and resilience should be integrated into interventions aimed at improving mental health and overall well-being among IBS patients.

### Implications

The findings of the present study have important implications for clinical practice and patient education in IBS. Given that cognitive flexibility influences psychological well-being both directly and indirectly through resilience, interventions that explicitly target flexible cognitive responding and adaptive problem solving may be particularly beneficial. This interpretation is supported by evidence from cognitive-behavioral therapy (CBT) research, particularly the study by Lackner et al. [20], which demonstrated that improvements in cognitive flexibility following CBT were associated with reductions in IBS symptom severity, abdominal pain, and improvements in quality of life. Enhancing patients’ capacity to shift perspectives, reinterpret symptoms, and respond flexibly to changing bodily and situational demands may strengthen perceived controllability over symptoms and foster resilience. In turn, greater resilience may buffer the psychological impact of chronic gastrointestinal symptoms by reducing distress, supporting emotional regulation, and improving overall quality of life.

These findings underscore the value of integrative, multidisciplinary approaches involving psychologists, gastroenterologists, and health educators. CBT-oriented interventions, mindfulness-based strategies, and structured problem-solving and stress-management programs may help patients develop flexible coping repertoires and sustain adaptive functioning over time. Educational workshops and support programs that emphasize active self-management, cognitive adaptability, and resilience-building can empower patients to engage more effectively with their condition, thereby supporting holistic, patient-centered care that addresses both psychological well-being and symptom burden.

### Limitations and direction for future research

Some limitations should be considered when interpreting these results. First, the cross-sectional design of this study did not allow for causal inferences, as it did not account for changes in cognitive flexibility, resilience, and psychological well-being over time. Longitudinal or experimental studies are needed to better understand the direction and stability of these relationships. Second, the sample was limited to IBS patients from the city of Isfahan, which may restrict the generalizability of the findings to other cultural, geographical, or clinical contexts. Third, the reliance on self-report measures may have introduced social desirability bias or inaccuracies in participants’ self-perceptions.

Fourth, an important limitation concerns the exclusion of individuals with severe psychological disorders. While this exclusion was necessary to focus on the primary psychological constructs without confounding effects of comorbid psychopathology, it inherently reduces the generalizability of the findings. Given that severe psychological disorders such as anxiety and depression are highly prevalent among IBS patients, the results may not fully represent this broader clinical population.

Fifth, while the Rome IV criteria were used for diagnosis, potential confounding factors such as IBS subtype (e.g., IBS-D, IBS-C), specific dietary habits, or medication use were not controlled for, which could influence the psychological variables measured. Additionally, potential confounding factors such as illness severity, comorbid psychological conditions, and lifestyle variables were not controlled for, which could influence the observed relationships.

Future research should aim to replicate these findings with larger, more diverse samples and employ longitudinal designs to track changes over time. It would also be valuable to incorporate objective measures of cognitive flexibility and resilience, as well as to explore the effectiveness of targeted interventions such as cognitive training or resilience-building programs in improving psychological well-being among IBS patients. Future studies should also include individuals with comorbid anxiety and depression to enhance external validity and provide a more accurate reflection of real-world IBS populations. Furthermore, research should investigate how these psychological constructs interact with biological markers (e.g., gut microbiome, inflammatory markers) and IBS subtypes to provide a more holistic biopsychosocial model of the condition. Expanding this line of research to other chronic illness populations could further clarify the broader applicability of these psychological mechanisms in health and disease management.

## Data Availability

Data will be provided upon reasonable request.

## Abbreviations

AGFI: Adjusted Goodness of Fit Index
CBT: Cognitive Behavioral Therapy
CFI: Comparative Fit Index
CMIN/DF: Chi-square to degrees of freedom ratio
COFI: Cognitive Flexibility Inventory
IBS: Irritable bowel syndrome
GFI: Goodness of Fit Index
IFI: Incremental Fit Index
NFI: Normed Fit Index
NNFI: Non-Normed Fit Index
PCLOSE: P-value for closeness of fit
PCFI: Parsimonious Comparative Fit Index
PNFI: Parsimonious Normed Fit Index
PWBS: Psychological Well-Being Scale
RMSEA: Root Mean Square Error of Approximation
SEM: Structural Equation Modeling
